# Correlating metabolic and anatomic responses of primary lung cancers to radiotherapy by combined F-18 FDG PET-CT imaging

**DOI:** 10.1186/1748-717X-2-18

**Published:** 2007-05-23

**Authors:** Ching-yee O Wong, Joseph Schmidt, Jeffery S Bong, Suyra Chundru, Larry Kestin, Di Yan, Inga Grills, Marianne Gaskill, Vincent Cheng, Alvaro A Martinez, Darlene Fink-Bennett

**Affiliations:** 1 Nuclear Medicine, William Beaumont Hospital, Royal Oak, Michigan, USA; 2 Radiology, Michigan State University College of Human Medicine, Lansing, Michigan, USA; 3 Radiation Oncology, William Beaumont Hospital, Royal Oak, Michigan, USA

## Abstract

**Background:**

To correlate the metabolic changes with size changes for tumor response by concomitant PET-CT evaluation of lung cancers after radiotherapy.

**Methods:**

36 patients were studied pre- and post-radiotherapy with^18^FDG PET-CT scans at a median interval of 71 days. All of the patients were followed clinically and radiographically after a mean period of 342 days for assessment of local control or failure rates. Change in size (sum of maximum orthogonal diameters) was correlated with that of maximum standard uptake value (SUV) of the primary lung cancer before and after conventional radiotherapy.

**Results:**

There was a significant reduction in both SUV and size of the primary cancer after radiotherapy (p < 0.00005). Among the 20 surviving patients, the sensitivity, specificity, and accuracy using PET (SUV) were 94%, 50%, 90% respectively and the corresponding values using and CT (size criteria) were 67%, 50%, and 65% respectively. The metabolic change (SUV) was highly correlated with the change in size by a quadratic function. In addition, the mean percentage metabolic change was significantly larger than that of size change (62.3 ± 32.7% vs 47.1 ± 26.1% respectively, p = 0.03)

**Conclusion:**

Correlating and incorporating metabolic change by PET into size change by concomitant CT is more sensitive in assessing therapeutic response than CT alone.

## Background

Positron Emission Tomography-Computed Tomography (PET-CT) imaging using [fluorine-18] fluorodeoxyglucose (^18^F-FDG) with CT attenuation and anatomical mapping has been widely used clinically in lung cancer diagnosis and treatment evaluation [1]. PET has been shown to stage lung cancers more accurately than CT scanning and provide high-impact and powerful prognostic stratification in staging newly diagnosed non-small cell lung cancers [2]. PET-CT offers a promising tool in both radiation treatment planning and response evaluation of radiotherapy by (a) quantifying the high metabolic rate among various cancer types in metabolizing serum glucose using ^18^F-FDG as its analog tracer in PET scanning [3] and (b) the potential ability of the concomitant mapping CT to measure the changes in tumor size. Mathematically, the glucose metabolic rate is calculated using the three-compartment model of ^18^F-FDG tracer kinetics [4,5]. The common measurement used by PET is the standard uptake value (SUV). This is defined by tumor activity per dose injected per body mass, which is proportional to the glucose metabolic rate within the normal range of serum glucose concentration [6,7].

The metabolic response is defined by the percentage change of post-radiotherapy SUV from the pre-radiotherapy (RTx) SUV as:

ΔM = (SUV_post-RTx_/SUV_pre-RTx _- 1) × 100%

According to the European Organization for Research and Treatment of Cancer (EORTC), metabolic response is characterized as a SUV reduction by at least 25% or ΔM < -25% [8]. Non-responders are classified as ΔM ≥ -25% [5]. Similar criteria for size changes has been proposed by the RECIST [9]. But both the metabolic and the size changes may have a continual spectrum. The purpose of the study was to investigate the correlation between changes of SUV of primary lung tumors following radiotherapy using ^18^F FDG PET-CT imaging with changes in tumor size measured on the concomitant CT.

## Methods

### Patient and radiation treatment

Thirty-six patients (15 males, 21 females), at a mean age of 64 ± 11 years, with primary lung cancers (16 adenocarcinoma, 11 non-small cell cancers, 4 squamous cell cancers and 5 small cell cancers) treated with radiotherapy with pretreatment dedicated contrast CT, F-18 FDG PET-CT and post-treatment PET-CT were included. Baseline pre-radiotherapy PET-CT was performed before any treatment, followed by post-radiotherapy PET-CT at a median of seventy-one days. All patients were considered either surgically or medically inoperable, and thus treated with radiotherapy using conventional protocols. All except in two patients with stage IA medically inoperable non-small cell lung cancer were also treated with standard chemotherapy. The clinical data is summarized in Table 1. The small cell lung cancers were treated with radiotherapy dose of 45 Gy in 1.5 Gy increments twice a day at 6-hour intervals or 50.4–54.0 Gy in 1.8 Gy fractions daily. The non-small cell lung cancers were treated with radiotherapy dose of 63 Gy in 1.8 Gy fractions once daily. The actual radiotherapy doses ranged from 60 – 66 Gy if given in 2 Gy fractions or 59.4 – 64.8 Gy if in 1.8 Gy fractions daily. Those medically inoperable patients with solitary tumor without nodal disease were treated with standard fractionated radiation alone might receive the radiation dose up to 70 Gy in 2 Gy fractions daily. All of the patients were followed clinically and radiographically after a mean period of 342 days for assessment of local control or failure rates.

**Table 1:** Clinical data

**Patient**	**Age**	**Sex**	**Tumor type**	**Tumor (T)**	**Node (N)**	**Metastasis (M)**	**Stage**	**Chemotherapy**
1	71	F	Adenocarcinoma	1	0	0	IA	No
2	63	F	Adenocarcinoma	2	1	0	IIB	Yes
3	68	M	Adenocarcinoma	3	0	0	IIB	Yes
4	62	M	Adenocarcinoma	2	2	0	IIB	Yes
5	50	F	Adenocarcinoma	3	2	0	III	Yes
6	74	M	Adenocarcinoma	2	2	0	IIIA	Yes
7	68	F	Adenocarcinoma	2	2	0	IIIA	Yes
8	69	M	Adenocarcinoma	3	1	0	IIIA	Yes
9	76	F	Adenocarcinoma	1	2	0	IIIA	Yes
10	34	F	Adenocarcinoma	4	1	0	IIIB	Yes
11	57	M	Adenocarcinoma	4	3	0	IIIB	Yes
12	75	M	Adenocarcinoma	1	3	0	IIIB	Yes
13	36	M	Adenocarcinoma	4	2	1	IV	Yes
14	73	F	Adenocarcinoma	3	3	1	IV	Yes
15	37	M	Adenocarcinoma	2	3	1	IV	Yes
16	58	F	Adenocarcinoma	4	0	1	IV	Yes
17	67	F	Non-small cell	1	1	0	IIA	Yes
18	69	F	Non-small cell	1	1	0	IIA	Yes
19	56	F	Non-small cell	4	1	0	IIIB	Yes
20	57	F	Non-small cell	3	3	1	IV	Yes
21	71	F	Non-small cell	1	0	0	IA	No
22	68	M	Non-small cell	2	0	0	IB	Yes
23	78	M	Non-small cell	4	2	0	IIIB	Yes
24	68	M	Non-small cell	3	3	0	IIIB	Yes
25	64	F	Non-small cell	4	0	0	IIIB	Yes
26	68	M	Non-small cell	4	3	1	IV	Yes
27	56	F	Non-small cell	2	3	1	IV	Yes
28	68	F	Squamous/Small cell	1	0	0	IA/limited	Yes
29	59	F	Squamous cell	3	0	0	IIB	Yes
30	65	F	Squamous cell	3	2	0	IIIA	Yes
31	76	M	Squamous cell	2	3	0	IIIA	Yes
32	77	F	Small cell	1	0	0	limited	Yes
33	74	F	Small cell	2	1	0	limited	Yes
34	62	F	Small cell	3	0	0	limited	Yes
35	53	M	Small cell	3	2	0	limited	Yes
36	60	M	Small cell	2	3	0	limited	Yes

### Imaging Technique

Imaging was obtained by a dedicated 16-slice body PET-CT scanner (GE Discovery DST, GE Medical Systems, Milwaukee, WI, USA). All patients with four-hour fasting before the examination received an average of 555 MBq ^18^FDG intravenous injections. PET images were obtained one hour after injection. The PET images were obtained at each bed position for 3 minutes with 6–8 beds to cover the entire body. The PET images were obtained using a two-dimensional high-sensitivity mode with an axial field of view of 15 cm in a 256 × 256 matrix. A 3-slice overlap was utilized between the bed positions. The PET images were reconstructed iteratively on a 128 × 128 matrix using ordered-subsets expectation maximization algorithm for 30 subsets and two iterations, with a 7.0-mm post-reconstruction filter. In-plane resolution of 6.2 mm and axial resolution of 5.0 mm was obtained. Concomitant CT data was used for attenuation correction of all PET images in the quantitative analysis of SUV. The CT component of image acquisition used the following imaging parameters: 140 kVp, 120–200 mA, 0.8 seconds per CT rotation, pitch 1.75:1, detector configuration of 16 × 1.25 mm, 3-mm slice thickness with oral contrast only.

### Image Evaluation and Analysis

Image analysis for tumors before and after therapy was performed by independent PET and CT readers. PET and CT images were also merged (fusion analysis) for functional and anatomic correlation. CT-PET images were displayed on AW/Xeleris and Medview workstations (General Electric Medical Systems, Milwaukee, WI, USA and Medimage, Ann Arbor, MI, USA). The pre- and post-radiotherapy SUV was calculated using the following formula:

SUV = lung cancer activity/(dose/lean body mass)

**Figure 1:**
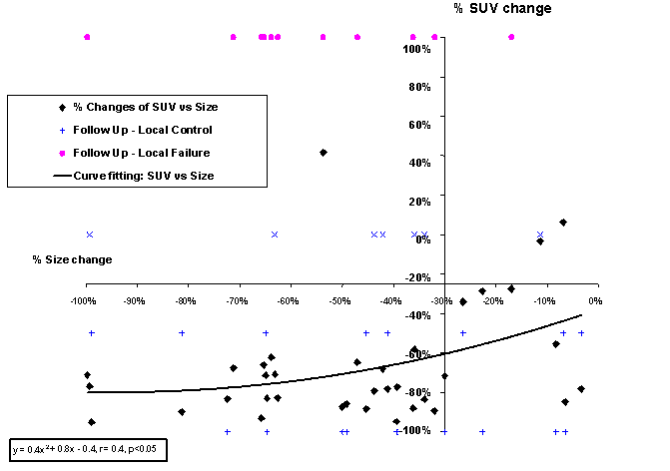
The percentage changes of size versus SUV with the axes cross the metabolic response line of -25% and anatomical response line of -30%. Legends, SUV = standard uptake value,  Plus sign = local control, cross = deceased, circle = local failure on follow up.

The maximum SUV (SUV_max_) was obtained by selecting volumetric regions of interest (VOIs) within the primary cancer site to include all tumor tissue but not any non-tumor tissue with potentially higher SUV than that of the tumor. The glucose concentration was also recorded for each patient before the injection of the F-18 FDG radiotracer in each PET scan. In addition, the two longest orthogonal diameters (Φ) of the primary tumor were measured on the CT component of PET-CT for each patient in lung window with validation by phantom studies [10]. The percentage of change in the sum of the two longest orthogonal diameters (Φ) was calculated as:

ΔΦ = (Φ_post-RTx_/Φ_pre-RTx _- 1) . 100%

and graphed with ΔM to correlate SUV change with size change for all patients (Fig. 1). Finally the magnitude of response measured by PET and CT was compared with clinical outcome using criteria at -25% and -30% respectively from EORTC [8] and RECIST [9] for metabolic and anatomical response. Statistical analysis was performed by SPSS, (SPSS Inc, Chicago, IL, USA) and a p-value < 0.05 was considered significant in all tests.

## Results

There was a significant difference in baseline SUV (15.8 ± 7.3) when compared to post-radiotherapy SUV (4.6 ± 3.9) (p < 0.00005). Tumor response was significantly evident by the change in size of the primary tumor from 8.1 ± 4.4 cm to 4.2 ± 2.2 cm before and after radiotherapy (p < 0.00005). The mean percentage metabolic change in SUV was 62.3 ± 32.7%, which was larger than the mean percentage change in size of 47.1 ± 26.1% (p = 0.03). The ΔM (SUV) significantly correlated with the ΔΦ (size) of the primary tumors by a quadratic function (Fig. 1, p < 0.05). The majority of the treated tumors were positioned within the tumor response quadrant by CT and PET response lines of -30% and -25%, respectively (Fig. 1, left lower quadrant), suggesting a fundamental effect on glucose metabolism and tumor size due to treatment.

Among the 20 surviving patients (Tables 2 and 3), the sensitivity, specificity and accuracy by PET metabolic response criteria in predicting the response using the gold standard of long term clinical and radiographic follow-up (Fig. 1) were 94%, 50%, 90% versus the corresponding values of 67%, 50%, and 65% by CT size criteria, respectively. The percentage SUV changes after radiotherapy was more sensitive and accurate than that of size change in predicting local control status (p = 0.02 and 0.03 respectively) (Tables 2 and 3) although the specificity was similar (p = ns).

**Table 2:** CT and local control status

**N = 20**	**CT Response**	**CT non-response**
**Local control**	12	6
**Local failure**	1	1

**Table 3:** PET and local control status

**N = 20**	**PET Response**	**PET non-response**
**Local control**	17	1
**Local failure**	1	1

Although the quadratic curve fitting of the data suggested the general non-linear correlation of the response by PET and CT (p < 0.05), the correlation in the metabolic and anatomic agreement zone was quite linear (left lower quadrant of Fig 1). This might be explained by cellular death that would ultimately lead to reduced metabolic activity and also eventual reduction in tumor size or tumor load. The data of PET-CT disagreement zone (right lower quadrant of Fig. 1) suggested that PET was superior to CT in identifying the group of patients who were misclassified by CT to be non-responders after radiotherapy using the long term follow-up as the gold standard. Figure 1 also demonstrated the observation that there was one patient with great shrinkage of tumor size, but no reduction of the metabolism to the required response level (left upper quadrant of Fig. 1). This patient was found later to be a true non-responder on follow-up.

## Discussion

Combined PET-CT is now gradually replacing single modality PET scan for diagnostic and staging evaluation of lung cancers. PET-CT is emerging as a tool for radiation treatment planning and monitoring of malignancies [10,11]. But the visual interpretation of PET-CT images is still dominating the oncologic diagnosis and treatment evaluation. The semi-quantitative SUV analysis not only separates the mean SUV values of benign versus malignant tissue, but also is a simple representation of the underlying tumor metabolism [7]. The size changes measured by the CT component depend primarily on the tumor shrinkage due to cellular death [12]. However, the size change may be affected by cystic, necrotic, fibrotic or hemorrhagic change within the tumor [12]. Without accurate respiratory gating during CT (4-D CT), the size measurement may be altered by respiratory motion. Thus, the impartial and dimensionless nature of quantitative measurement of maximum SUV change makes it a valuable adjunct to visual analysis of the PET component in the PET-CT imaging, especially without another dimension from respiratory gating.

The current study investigated responses measured by PET-CT, which yielded the combined effects of change in metabolism and physical size to reflect the change in underlying tissue after radiotherapy. Moreover, the metabolic measurement of radiotherapy response by PET (SUV) correlated with the traditional change in size on the concomitant CT during PET-CT imaging especially on the combined responding zone (Fig. 1), which was the main focus of treatment evaluation. Due to some discrepancy in the magnitude of responses between the biologic and physical criteria, PET imaging will impact clinically when metabolic response (SUV change) differs from change in size.

The primary factor for variations in SUV after treatment was the reduction of metabolism due to cellular death or less likely, in case of an effective treatment, augmentation of metabolism due to tumor progression. The contribution of this current study was to investigate the correlation and impact of metabolic change by the PET component using SUV with size change measured by CT. This incorporation enables comprehensive anatomolecular criteria for treatment response. The results demonstrated that the findings of PET and traditional CT response were correlated to reflect the anticipated clinical treatment effects.

The study measured the SUV change by searching the entire volume of interest to get the maximum SUV. The PET component measured metabolic activity in an averaged respiratory cycle and thus was less affected by respiratory motion than the size change measured by non-4D CT used in the current PET-CT. The combined changes of SUV (by PET) and size measurements (by CT) may potentially compliment each other. This is particularly important biologically when the size of tumor does not shrink quickly or significantly in post-treatment CT scans. The results, however, showed less than expected deviations of SUV versus size change with PET-CT imaging. This might be related to the fact that patients were scanned about 71 days after radiation, which reduced if not eliminated, the potential effects of post-radiation inflammation and had given enough time for the tumor to shrink. In addition, the study demonstrated that it would be rare to bring the post-radiotherapy SUV or size to absolute zero as there might have inflammatory cells and/or some granulation/scar tissue present at the original tumor site after treatment, as studied previously [13].

The current study shows that a comprehensive metabolic evaluation of tumor response may be obtained by PET supplemented with the change in size evaluated by the CT component (Figure 1) resulting in the multi-dimensional multi-modality evaluation. This may play a vital role in the trend towards biologic imaging for tumor response evaluation after radiotherapy, with potential prognostic implications [14,15]. There is ample evidence of prognostic implications of PET scan in other tumors such as lymphoma [16,17] and its prediction of relapse [18,19]. Moreover, in the evaluation of the response to the treatment for lymphoma, there is growing interest in patient response early during treatment [20,21], just like PET for assessing neo-adjuvant treatment for lung cancers [14,15]. With the introduction of the concept of maximum SUV, which is a dimensionless quantity incorporating into size change, it appears that the first step in improvement of PET-CT evaluation has been achieved.

In summary, the maximum SUV change is a useful parameter in oncologic PET-CT measurement for comparison and monitoring of treatment response, especially in a situation when size change is variable. The changes of SUV and size before and after radiotherapy (Figure 1) allow additional dimension to the traditional single modality treatment monitoring evaluation using CT alone. The four quadrants formed by PET and CT response lines (as illustrated in Figure 1) reveal the four possible combinations or scenarios of metabolic and anatomical responses. While this method is currently validated in various primary lung cancers, the specific numerical results may be generalized to other cancers. This is an important consideration in view of the emerging biologic imaging guided adaptive radiotherapies.

## Conclusion

The correlation between changes in SUV and size using combined PET-CT imaging shows promise in the improved treatment response parameters. The study showed that incorporating metabolic change by PET into concomitant size change by CT is more sensitive and accurate in predicting local control than CT alone which may have a significant impact in evaluation of response for different types of cancers.
